# A rare case report of celiac disease: Hypoglycemic coma in a nondiabetic patient

**DOI:** 10.1097/MD.0000000000043029

**Published:** 2025-06-27

**Authors:** Yi-Hsin Lin, Hsuan Huang

**Affiliations:** a Division of Endocrinology and Metabolism, Department of Internal Medicine, Taiwan Adventist Hospital, Taipei, Taiwan; b Division of Pediatric Surgery, Department of Surgery, Mackay Memorial Hospital, Taipei, Taiwan.

**Keywords:** celiac disease, hypoglycemic coma, non-diabetes

## Abstract

**Rationale:**

Celiac disease (CD) is an autoimmune disorder triggered by gluten ingestion in genetically predisposed individuals, leading to small intestinal damage and malabsorption. While classic gastrointestinal symptoms such as diarrhea and weight loss are well-documented, extraintestinal manifestations, including neurological and endocrine abnormalities, are increasingly recognized. However, hypoglycemic coma as a presenting feature of CD is exceedingly rare, particularly in nondiabetic individuals.

**Patient concerns:**

A 24-year-old Caucasian male, recently relocated to Taiwan for academic studies, presented with progressive weight loss, chronic diarrhea, generalized weakness, and dizziness over 3 months. His symptoms culminated in a hypoglycemic coma, prompting emergency medical attention. Laboratory evaluations ruled out common causes of hypoglycemia, and further assessment was pursued to identify an underlying etiology.

**Diagnoses:**

Initial serological testing revealed a positive anti-deamidated gliadin peptide immunoglobulin A despite a negative anti-tissue transglutaminase immunoglobulin A. Subsequent endoscopic biopsy confirmed CD with Marsh type I histopathological changes.

**Interventions and outcomes:**

The patient was prescribed a strict gluten-free diet, leading to symptom resolution, weight recovery, and stabilization of blood glucose levels over follow-up.

**Lessons:**

This case underscores the importance of considering CD in the differential diagnosis of unexplained hypoglycemia, particularly in individuals with concurrent gastrointestinal symptoms. It also highlights the potential challenges of living with undiagnosed CD in regions where gluten labeling and dietary awareness may be inconsistent. Early recognition and dietary intervention are crucial in preventing severe complications associated with CD.

## 1. Introduction

Celiac disease (CD), a chronic autoimmune disorder triggered by gluten ingestion in genetically predisposed individuals, has a global prevalence of approximately 1%, though its presentation varies widely.^[1,2]^ The pathophysiology of CD involves immune-mediated damage to the small intestinal mucosa, often leading to malabsorption and systemic manifestations. While classic symptoms such as chronic diarrhea, weight loss, and iron-deficiency anemia are well-documented, atypical presentations, including neurological, dermatological, and endocrine abnormalities, are increasingly recognized.^[2]^ Among these, hypoglycemic events are exceedingly rare and often underreported in the absence of diabetes or other metabolic disorders.^[3]^

CD had been considered uncommon in Asia for a long time. Several studies suggested that, in the Indian subcontinent and Middle East countries, CD is present and as prevalent as in Western countries.^[4,5]^ Outside these Asian regions, the information about the epidemiology of CD is still lacking or largely incomplete for different and variable reasons, in which the prevalence of CD is very likely to be underestimated.^[6–10]^ Several factors may, to a different extent, contribute to CD underdiagnosis (and, thus, underestimation of its epidemiological burden), including the poor disease awareness among physicians and/or patients, limited access to diagnostic resources, inappropriate use or interpretation of the serological tests, absence of standardized diagnostic and endoscopic protocols, and insufficient expertise in histopathological interpretation.^[5]^

This report highlights a unique case of hypoglycemic coma as the presenting feature of undiagnosed CD in a young, non-diabetic adult. By exploring the clinical trajectory and diagnostic challenges of this case, we aim to underscore the importance of considering CD in the differential diagnosis of unexplained hypoglycemia, especially in patients with concurrent gastrointestinal symptoms or malabsorption syndromes.

## 2. Case report

We present the case of a 24-year-old Caucasian male graduate student from the United States who arrived in Taiwan 6 months prior for academic studies. He reported being in good health with no history of chronic illnesses. However, 3 months after his arrival, he developed a gradual loss of appetite and persistent diarrhea, resulting in a 7-kg weight loss over 3 months (from 80 to 73 kg). He frequently experienced weakness, dizziness, and general malaise. The patient’s condition worsened, culminating in a brief loss of consciousness at home lasting approximately 1 minute. Upon arrival at the emergency department via ambulance, paramedics measured his blood glucose level, which was critically low at 47 mg/dL. He was diagnosed with a hypoglycemic coma and admitted for further evaluation and management.

**Figure 1.**
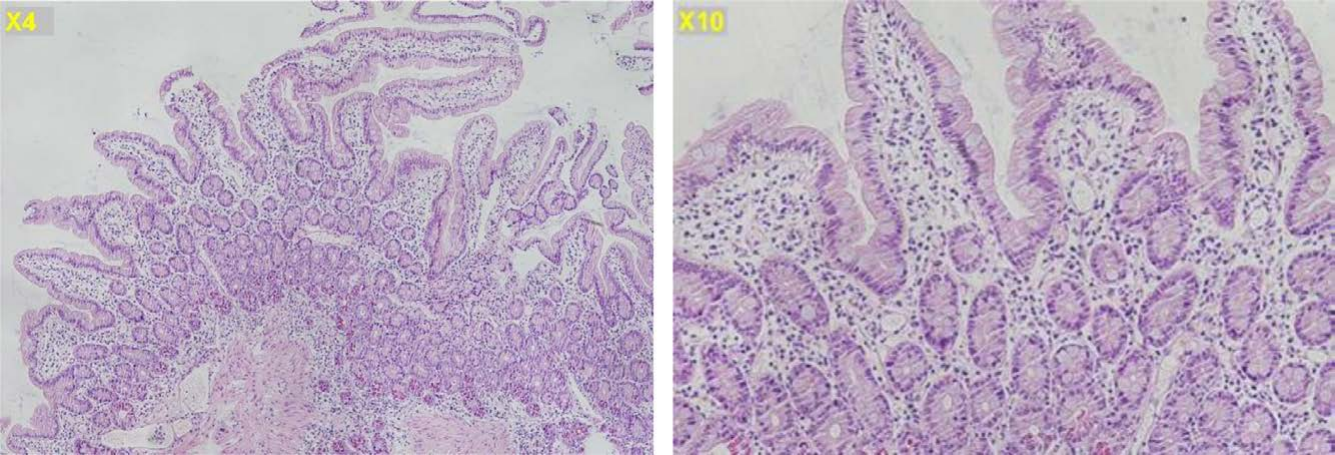
Endoscopic biopsy of the duodenal intestine revealed normal villi structure and no crypt hyperplasia, but mild lymphocytic infiltration, which was consistent with celiac disease, Marsh classification type I histopathological changes.

A comprehensive diagnostic workup was conducted to identify the underlying cause of his hypoglycemia, chronic diarrhea, and malabsorption. During the medical history review, it was revealed that his older sister had been diagnosed with CD. This familial history raised clinical suspicion of CD as a potential underlying cause. The initial laboratory tests revealed no abnormality, except for positive antinuclear antibody (1:160X [reference value: negative < 1:40X]). Following serological tests revealed negative anti-tissue transglutaminase immunoglobulin A (IgA) (1 [reference value: negative: <15 U/mL, positive: 15 U/mL or greater]), but positive anti-deamidated gliadin peptide IgA (118 [reference value: negative: <15 U/mL, positive: 15 U/mL or greater]; Table 1). Endoscopic biopsy of the small intestine confirmed CD, demonstrating Marsh classification type I histopathological changes (Fig. 1).

**Table 1:** Laboratory tests on the arrival and further workup.

	Result	Reference value
Hemoglobin	14.5 g/dL	13.0–18.0
White blood cell	7.0 × 1000/μL	3.8–10.0
Platelet	174 × 1000/μL	140.0–450.0
Creatinine	0.93 mg/dL	0.70–1.30
S-GPT	15 IU/L	7–52
C-reaction protein	<0.1 mg/dL	<1.0
E.S.R. (1 h)	1.0 mm/h	0.0–15.0
HbA1c	4.8%	4.0–6.0
Blood sugar	87 mg/dL	70–120
C-peptide	1.40 ng/mL	1.06–3.53
Total cholesterol	156 mg/dL	<200
Triglyceride	52 mg/dL	<150
High density lipoprotein-cholesterol	57 mg/dL	≥50
Low density lipoprotein-cholesterol	97 mg/dL	<130
Sodium	140.0 mmol/L	136.0–145.0
Potassium	3.8 mmol/L	3.5–5.1
Calcium	10.0 mg/dL	8.6–10.3
Magnesium	2.1 mg/dL	1.9–2.7
Albumin	5.1 g/dL	3.5–5.7
Free T4	0.85 ng/dL	0.66–1.17
TSH	1.67 μIU/mL	0.38–5.33
Anti-TPO	11 IU/mL	<34
Anti-thyroglobulin antibody	12 IUmL	<115
Anti-SSA/Ro	60 Au/mL	<100, negative
Anti-SSB/La	10 Au/mL	<100, negative
Anti-Smith-Ab	53 Au/mL	<100, negative
Anti-nRNP	54 Au/mL	<100, negative
Anti-SCL70	23 Au/mL	<100, negative
Anti-JO-1	54 Au/mL	<100, negative
Antinuclear antibody	1:160X, positive	1:40X, negative
Rheumatoid factor	Negative	Negative
Cortisol (8 am)	14.3 pg/dL	4.8–19.5
Vit-B12	316 pg/mL	197–771
Folic acid	6.82 ng/mL	3.89–26.8
Anti-tissue transglutaminase IgA	1 U/mL	<15, negative
Anti-deamidated gliadin peptide IgA	118 U/mL	<15, negative

anti-nRNP = anti-nuclear ribonucleoprotein antibodies, anti-SSA/Ro = anti-Sjögren’s syndrome related antigen A autoantibodies, anti-SSB/La = anti-Sjögren’ s syndrome-related antigen B autoantibodies, anti-TPO = anti-thyroid peroxidase antibody, HbA1c = hemoglobin A1c, IgA = immunoglobulin A, S-GPT = serum-glutamate pyruvate transaminase, TSH = thyroid stimulating hormone, Vit-B12 = vitamin B12.

The patient was prescribed a gluten-free diet and discharged with dietary counseling. Follow-up evaluations showed a gradual resolution of his symptoms, restoration of physical strength, and recovery of his original weight. This case emphasizes the need to consider CD in the differential diagnosis of hypoglycemic coma, even in non-diabetic individuals.

## 3. Discussion

CD is a multifaceted autoimmune disorder with a wide spectrum of clinical presentations, ranging from classic malabsorption symptoms to atypical extraintestinal manifestations.^[11,12]^ Hypoglycemia is an uncommon presentation of CD. Most cases of hypoglycemia in CD are associated with coexisting conditions such as type 1 diabetes mellitus, where impaired glucose regulation and malabsorption may exacerbate metabolic instability.^[13–16]^ CD and other endocrine disease, such as Addison’s disease, or hypothyroidism should be considered in children and adolescents with unexplained hypoglycemia.^[17]^ However, reports of hypoglycemia in non-diabetic individuals, such as the present case, remain exceedingly rare, making this case noteworthy.

The pathogenesis of hypoglycemia in CD remains speculative but is likely multifactorial.^[18]^ Chronic malabsorption resulting from gluten-induced small intestinal damage may lead to deficiencies in macronutrients and micronutrients critical for glucose metabolism. Severe energy imbalance, secondary to prolonged diarrhea and reduced dietary intake, could predispose patients to hypoglycemia. Moreover, altered intestinal absorption of carbohydrates might impair glucose homeostasis, particularly in prolonged fasting states.^[19]^ These possible mechanisms align with our patient’s clinical trajectory, where prolonged diarrhea, weight loss, and reduced oral intake likely precipitated hypoglycemia.

The diagnosis of the patient was established through a combination of serological testing and endoscopic biopsy findings, consistent with the World Gastroenterology Organization guidelines for CD.^[20]^ The initial negative anti-tissue transglutaminase IgA result highlights the need for comprehensive serological evaluation, including anti-deamidated gliadin peptide IgA, especially in cases of IgA deficiency or atypical presentations. Histopathological confirmation using the Marsh classification remains the cornerstone of diagnosis in such cases, providing definitive evidence of gluten-induced enteropathy.^[21]^ The patient’s remarkable clinical recovery following the initiation of a gluten-free diet emphasizes the transformative potential of dietary management in CD. Strict adherence to a gluten-free diet not only resolves gastrointestinal symptoms but also alleviates systemic complications, which emphasize the necessity of early diagnosis and intervention to prevent severe morbidity associated with CD.^[22]^

Based on the World Gastroenterology Organization guidelines and literature reviews, the lower prevalence of CD in Asian populations compared to Western or Caucasian populations can be attributed to several key factors.^[20,23]^ Firstly, genetic factors: CD is strongly associated with HLA-DQ2 and HLA-DQ8 alleles. These genetic markers are significantly less common in many Asian populations, such as in Japan, Korea and the Far East, compared to Western populations. The presence of these alleles is a necessary but insufficient condition for developing CD, further reducing its prevalence in populations with a lower frequency of these genes.^[24]^ Secondly, dietary patterns: traditional Asian diets primarily rely on rice and other gluten-free grains, with less emphasis on wheat, barley, or rye – key sources of gluten. Lower gluten exposure reduces the environmental trigger necessary for the development of CD.^[25,26]^ Thirdly, environmental factors: the “hygiene hypothesis” theorizes that decreased exposure to pathogens might increase the risk of autoimmune conditions like CD. This factor may contribute to its lower prevalence in regions with higher pathogen exposure during early childhood.^[27]^ Finally, diagnostic and awareness gaps: a lack of awareness about CD and limited access to diagnostic tools in some Asian countries may result in underdiagnosis, further contributing to the perception of a lower prevalence. Moreover, the lack of clear gluten labeling and ambiguous ingredient information in many Asian foods could plausibly contribute to the patient’s inadvertent gluten consumption, exacerbating his condition and leading to the hypoglycemic episode.^[5]^

This case highlights a significant challenge faced by individuals with undiagnosed CD when living in regions where gluten labeling is inconsistent or absent. Asian cuisines often incorporate gluten-containing ingredients such as soy sauce, wheat starch, or flour in a variety of dishes, many of which are not labeled as gluten-containing. Furthermore, gluten-free alternatives are less commonly available, and cross-contamination in food preparation is a frequent issue. For this patient, transitioning to a new dietary environment without knowledge of gluten’s hidden presence in many traditional Asian foods may have led to ongoing gluten exposure. This, in turn, perpetuated mucosal damage and malabsorption, ultimately contributing to the hypoglycemic episode. Such inadvertent gluten ingestion highlights the importance of clear labeling and education about gluten-containing ingredients, particularly for individuals with CD who travel or reside in areas with differing food regulations and dietary practices.^[28]^

## 4. Conclusion

This case adds to the growing body of literature emphasizing the diverse presentations of CD. Physicians should maintain a high index of suspicion for CD in patients with unexplained hypoglycemia, particularly when accompanied by signs of malabsorption. Early, comprehensive diagnostic evaluation diagnosis and timely dietary intervention are crucial to improving outcomes and reducing long-term complications.

## Acknowledgments

The authors would like to express their deepest gratitude to all the people who participated in this study. Our heartfelt thanks go to the hospital officials for their guidance and support for this study.

## Author contributions

**Conceptualization:** Yi-Hsin Lin, Hsuan Huang.

**Data curation:** Yi-Hsin Lin.

**Investigation:** Yi-Hsin Lin.

**Supervision:** Hsuan Huang.

**Validation:** Hsuan Huang.

**Writing – original draft:** Yi-Hsin Lin.

**Writing – review & editing:** Hsuan Huang.

## References

[1] Gujral N Freeman HJ Thomson AB. Celiac disease: prevalence, diagnosis, pathogenesis and treatment. World J Gastroenterol. 2012;18:6036–59. 23155333 10.3748/wjg.v18.i42.6036 PMC3496881

[2] Caio G Volta U Sapone A. Celiac disease: a comprehensive current review. BMC Med. 2019;17:142. 31331324 10.1186/s12916-019-1380-z PMC6647104

[3] Eland I Klieverik L Mansour AA Al-Toma A. Gluten-free diet in co-existent celiac disease and type 1 diabetes mellitus: is it detrimental or beneficial to glycemic control, vascular complications, and quality of life? Nutrients. 2022;15:199. 36615856 10.3390/nu15010199 PMC9824312

[4] Singh P Arora S Singh A Strand TA Makharia GK. Prevalence of celiac disease in Asia: a systematic review and meta-analysis. J Gastroenterol Hepatol. 2016;31:1095–101. 26678020 10.1111/jgh.13270

[5] Poddighe D Abdukhakimova D. Celiac disease in Asia beyond the Middle East and Indian subcontinent: epidemiological burden and diagnostic barriers. World J Gastroenterol. 2021;27:2251–6. 34040319 10.3748/wjg.v27.i19.2251 PMC8130036

[6] Bach JF. The hygiene hypothesis in autoimmunity: the role of pathogens and commensals. Nat Rev Immunol. 2018;18:105–20. 29034905 10.1038/nri.2017.111

[7] Corazza GR Andreani ML Biagi F. The smaller size of the “coeliac iceberg” in adults. Scand J Gastroenterol. 1997;32:917–9. 9299671 10.3109/00365529709011202

[8] Ivarsson A Persson LA Juto P Peltonen M Suhr O Hernell O. High prevalence of undiagnosed coeliac disease in adults: a Swedish population-based study. J Intern Med. 1999;245:63–8. 10095818 10.1046/j.1365-2796.1999.00403.x

[9] Riestra S Fernandez E Rodrigo L Garcia S Ocio G. Prevalence of coeliac disease in the general population of northern Spain. Scand J Gastroenterol. 2000;35:398–402. 10831263 10.1080/003655200750023967

[10] Volta U Bellentani S Bianchi FB. High prevalence of celiac disease in Italian general population. Dig Dis Sci. 2001;46:1500–5. 11478502 10.1023/a:1010648122797

[11] Fasano A. Clinical presentation of celiac disease in the pediatric population. Gastroenterology. 2005;128(4 Suppl 1):S68–73. 15825129 10.1053/j.gastro.2005.02.015

[12] Tarar ZI Zafar MU Farooq U Basar O Tahan V Daglilar E. The progression of celiac disease, diagnostic modalities, and treatment options. J Investig Med High Impact Case Rep. 2021;9:23247096211053702. 10.1177/23247096211053702 PMC8767653 34693776

[13] Hakami M Yafei S Hummadi A. Clinical characteristics and prevalence of celiac disease in a large cohort of type 1 diabetes from Saudi Arabia. Medicina (Kaunas). 2024;60:1940. 39768822 10.3390/medicina60121940 PMC11676682

[14] Alibrahim AR Al-Saleh YM Basahih TO. The prevalence of associated autoimmune diseases among adults with type 1 diabetes mellitus: a cross-sectional study. Cureus. 2022;14:e27190. 36039251 10.7759/cureus.27190 PMC9395765

[15] Mohn A Cerruto M Iafusco D. Celiac disease in children and adolescents with type I diabetes: importance of hypoglycemia. J Pediatr Gastroenterol Nutr. 2001;32:37–40. 11176322 10.1097/00005176-200101000-00012

[16] Khoury N Semenkovich K Arbeláez AM. Coeliac disease presenting as severe hypoglycaemia in youth with type 1 diabetes. Diabet Med. 2014;31:e33–6. 24805141 10.1111/dme.12488

[17] Zucchini S Tumini S Scaramuzza AE. Recommendations for recognizing, risk stratifying, treating, and managing children and adolescents with hypoglycemia. Front Endocrinol (Lausanne). 2024;15:1387537. 38894740 10.3389/fendo.2024.1387537 PMC11183505

[18] Bains K Kalra S Singh I. Prevalence and impact of malnutrition in hospitalizations among celiac diseases: a nationwide analysis. Cureus. 2023;15:e44247. 37772221 10.7759/cureus.44247 PMC10524785

[19] Barton SH Kelly DG Murray JA. Nutritional deficiencies in celiac disease. Gastroenterol Clin North Am. 2007;36:93–108, vi. 17472877 10.1016/j.gtc.2007.01.006

[20] Bai JC Ciacci C. World gastroenterology organisation global guidelines: celiac disease february 2017. J Clin Gastroenterol. 2017;51:755–68. 28877080 10.1097/MCG.0000000000000919

[21] Villanacci V Vanoli A Leoncini G. Celiac disease: histology-differential diagnosis-complications. A practical approach. Pathologica. 2020;112:186–96. 33179621 10.32074/1591-951X-157 PMC7931573

[22] Raiteri A Granito A Giamperoli A Catenaro T Negrini G Tovoli F. Current guidelines for the management of celiac disease: a systematic review with comparative analysis. World J Gastroenterol. 2022;28:154–75. 35125825 10.3748/wjg.v28.i1.154 PMC8793016

[23] Singh P Arora A Strand TA. Global prevalence of celiac disease: systematic review and meta-analysis. Clin Gastroenterol Hepatol. 2018;16:823–36.e2. 29551598 10.1016/j.cgh.2017.06.037

[24] Tamai T Ihara K. Celiac disease genetics, pathogenesis, and standard therapy for Japanese patients. Int J Mol Sci. 2023;24:2075. 36768398 10.3390/ijms24032075 PMC9916540

[25] El Khoury D Balfour-Ducharme S Joye IJ. A review on the gluten-free diet: technological and nutritional challenges. Nutrients. 2018;10:1410. 30279384 10.3390/nu10101410 PMC6213115

[26] Park J Kim HS. Rice-based gluten-free foods and technologies: a review. Foods. 2023;12:4110. 38002168 10.3390/foods12224110 PMC10670158

[27] Pes GM Bibbò S Dore MP. Coeliac disease: beyond genetic susceptibility and gluten. A narrative review. Ann Med. 2019;51:1–16. 10.1080/07853890.2019.1569254 PMC7857446 30739507

[28] Herrera-Quintana L Navajas-Porras B Vázquez-Lorente H. Celiac disease: beyond diet and food awareness. Foods. 2025;14:377. 39941971 10.3390/foods14030377 PMC11817883

